# Performance of DeepSeek-R1 and ChatGPT-5 in the Generation of North American Spine Society Clinical Guidelines for Adult Vertebral Compression Fractures: Comparative Study

**DOI:** 10.2196/87816

**Published:** 2026-07-10

**Authors:** Ruiyuan Chen, Yue Pan, Minghui Liang, Aobo Wang, Ziqian Ma, Yu Xi, Ning Fan, Shuo Yuan, Peng Du, Tianyi Wang, Lei Zang

**Affiliations:** 1 Department of Orthopedics, Beijing Chao-Yang Hospital, Capital Medical University, 5 JingYuan Road, Shijingshan District, Beijing, 100043, China, 86 51718688; 2 Department of Orthopedics, Beijing Mentougou District Hospital, Beijing, China

**Keywords:** artificial intelligence, ChatGPT, DeepSeek, large language model, North American Spine Society clinical guideline, vertebral compression fractures

## Abstract

**Background:**

Vertebral compression fractures (VCFs) impose a substantial clinical and health care burden, and their management relies on timely access to evidence-based guidelines. Large language models (LLMs) may help clinicians rapidly obtain guideline-related information, but their performance on VCF guidelines remains unclear.

**Objective:**

This study aimed to evaluate the performance of LLMs, including DeepSeek-R1 and ChatGPT-5, in generating responses consistent with VCF clinical guidelines.

**Methods:**

Using the 2024 North American Spine Society VCF clinical guidelines as the reference standard, 34 open-ended and 87 closed-ended questions were submitted to DeepSeek-R1 and ChatGPT-5. Four senior spine surgeons independently rated responses to both closed-ended and open-ended questions using a 5-point Likert scale for accuracy, consistency, self-awareness, and fabrication/falsification. For open-ended questions, comprehensiveness, clarity, and trust and confidence were additionally assessed. Subgroup analyses were performed by question type, recommendation grade, and VCF subtype, with direct comparisons between models.

**Results:**

A total of 726 responses were generated for 121 questions. For closed-ended questions, ChatGPT-5 and DeepSeek-R1 showed comparable performance in accuracy (*P*=.11), self-awareness (*P*=.10), and fabrication/falsification (*P*=.10). DeepSeek-R1 demonstrated better consistency than ChatGPT-5 for both closed-ended and open-ended questions (*P*<.001 and *P*=.001, respectively). For open-ended questions, the models differed significantly in comprehensiveness (*P*=.03) and trust and confidence (*P*=.02), but not in accuracy (*P*=.42), self-awareness (*P*=.22), fabrication/falsification (*P*=.64), or clarity (*P*=.48). Closed-ended questions generally outperformed open-ended questions. Responses to grade A-C recommendations outperformed grade I recommendations in accuracy, consistency, and fabrication/falsification (all *P*≤.001) but scored lower in self-awareness (*P*<.001). No significant differences were observed across VCF subtypes.

**Conclusions:**

Under a standardized clinician-oriented prompting condition, ChatGPT-5 and DeepSeek-R1 showed generally high but variable scores across evaluation dimensions, with important deficiencies remaining, particularly in interventional and surgical treatment recommendations and in questions linked to recommendation grade I. Because these findings were obtained in a controlled prompting setting, caution is warranted when extrapolating them to other query styles, clinical scenarios, or LLMs.

## Introduction

With population aging, vertebral compression fractures (VCFs) are imposing an increasingly serious challenge on public health systems worldwide [1-5]. VCFs frequently cause pain and spinal deformity and may increase the risk of age-adjusted mortality [4,6-8]. Statistically, approximately 1.5 million American adults are affected by VCFs each year, with an estimated annual health care cost of up to US $13.8 billion [9]. Therefore, comprehensive management of patients with VCFs remains a major challenge in spinal surgery and requires adherence to authoritative clinical guidelines and a standardized, multidimensional approach throughout the perioperative period. However, for busy clinicians, searching for the latest comprehensive diagnostic and treatment standards for VCFs poses a significant challenge, necessitating more accessible avenues to obtain reliable information.

The appearance of large language models (LLMs) opens up the possibility of addressing these challenges [10,11]. LLMs are trained on an extensive corpus of domain-specific text data from across the internet and have shown substantial potential in answering questions across a wide range of medical domains [12]. Research in the fields of ophthalmology and urology has demonstrated that LLMs can provide excellent clinical recommendations [13,14]. However, the accuracy of LLMs varied significantly when queried on spinal surgery topics, such as antibiotic prophylaxis, thromboembolism prophylaxis, low back pain, degenerative spondylolisthesis, and cervical radiculopathy [15-19]. Hence, it is important to examine how LLM performance varies across domains. However, to our knowledge, no study has investigated the performance of current LLMs on VCF. Meanwhile, previous studies were constrained by the timeliness of guidelines, resulting in an information gap between LLM training and guideline development [15,16,18,19]. The North American Spine Society (NASS) published its updated guidelines for the management of osteoporotic and neoplastic VCFs in 2024, providing an advantageous opportunity and reliable ground truth for evaluating current LLMs.

Therefore, using a QUEST (Quality of Information, Understanding and Reasoning, Expression Style and Persona, Safety and Harm, and Trust and Confidence)-aligned multidimensional evaluation framework adapted for guideline-based VCF assessment, this study evaluated DeepSeek-R1 and ChatGPT-5 responses to clinical questions and recommendation-based items derived from the updated 2024 NASS guidelines. The primary objective was to compare the multidimensional response quality and guideline concordance of the 2 models. The secondary objective was to determine whether model performance varied according to question type, recommendation grade, and VCF subtype. We hypothesized that performance would be higher for closed-ended questions and for recommendations supported by stronger evidence grades.

## Methods

### Overview

This study was a cross-sectional observational evaluation conducted using an adapted QUEST-aligned human evaluation framework and the STROBE (Strengthening the Reporting of Observational Studies in Epidemiology) reporting guideline (Checklist 1) [20,21].

### Ethical Considerations

This study was based entirely on publicly available NASS clinical guidelines and LLM-generated text outputs and did not involve human participants, patient data, biological specimens, or identifiable personal information. Therefore, institutional ethical review and informed consent were not required.

### Dataset Construction

Considering both authority and timeliness, this study included the latest NASS guidelines published in September 2024: “Evidence-Based Guidelines for Multidisciplinary Spine Care: Diagnosis and Treatment of Adults with Osteoporotic Vertebral Compression Fractures” and “Evidence-Based Clinical Guidelines for Multidisciplinary Spine Care: Diagnosis and Treatment of Adults with Neoplastic Vertebral Fractures” [22,23]. These guidelines included a total of 51 clinical questions across 7 sections: natural history, cost-effectiveness, clinical diagnosis, drug therapy, imaging diagnosis, interventional therapy, and surgical treatment. Seventeen clinical questions were excluded from this study because they were not accompanied by a recommendation grade and were instead answered with the statement, “A systematic review of the literature yielded no studies to adequately address this question.” The remaining 34 clinical questions were retained as open-ended questions. To further evaluate the ability of LLMs to address more detailed and specific issues, each recommendation statement was reformulated into 1 closed-ended recommendation-based item while preserving its original recommendation direction and substantive meaning. Because a single clinical question could contribute more than 1 closed-ended recommendation statement, a total of 87 closed-ended questions were ultimately generated. The guidelines used 4 recommendation grades: A (recommended), B (suggested), C (may be considered), and I (there is insufficient evidence to make a recommendation for or against).

### LLM Selection and Prompt Strategy

This study evaluated 2 advanced mainstream LLMs: ChatGPT-5 (OpenAI) and DeepSeek-R1 (DeepSeek). The main characteristics of these LLMs are detailed in Multimedia Appendix 1.

Given that prompt wording can influence the quality of LLM responses, we carefully designed a prompt based on published prompt engineering guidance. This prompt was intended to align the interaction with the clinical orientation and specialty-specific nature of the study task and to encourage evidence-based responses in a professional context: “Imagine you are an experienced spine surgeon with a knowledgeable background in the latest research in the field of VCFs. Please answer the following prompt based on evidence-based research: [Query]?” [24-26]. To ensure standardization of the evaluation framework, consistent prompts were used for both LLMs. Each prompt was input into each LLM 3 times. Each prompt was input into a new window in each LLM, and the answers were recorded verbatim. The memory setting and the “internet search” function were disabled to simulate a frozen-knowledge, zero-shot role-prompting condition [27,28]. The prompting and sorting process was performed by a spine surgeon (S1, with research experience in validating models using clinical practice guidelines) over a 3-day period in August 2025. All prompts based on the same question were repeated on the same day, thereby minimizing the impact of temporal intervals. All nonanonymous information, including model names, interface formatting, and any platform-generated disclaimers or warning messages, was removed before the materials were provided to the 4 evaluators, and the order of questions and models was randomized (Multimedia Appendix 2).

### Performance Evaluation

The primary evaluation construct in this study was guideline-concordant response quality. The evaluation outcomes were grouped into 4 main domains. The first domain, quality of information, included accuracy, consistency, and comprehensiveness. The second domain, expression style, included clarity. The third domain, safety, included self-awareness and fabrication and falsification. The fourth domain comprised trust and confidence. For both closed-ended and open-ended questions, knowledge accuracy and safety behaviors were evaluated [20,29]. For open-ended questions, we further evaluated comprehensiveness, expression style, and trust and confidence. Accuracy, self-awareness, fabrication and falsification, comprehensiveness, clarity, and trust and confidence were evaluated using a 5-point Likert scale based on the response to the first prompt for each LLM. The remaining repeated responses were used solely for consistency evaluation. Because no examples, prior conversational context, or memory were provided, this design reflected a zero-shot condition. However, because the prompt explicitly assigned the model the role of an experienced spine surgeon, we describe this framework as zero-shot role-prompting rather than a fully neutral baseline prompting condition. For consistency, the 3 responses generated from the same prompt in 3 separate new sessions were reviewed together, and each evaluator assigned one overall Likert score to the set based on the stability and uniformity of the responses across repeated zero-shot role-prompting runs. On the 5-point Likert scale, a score of 1 indicated clearly unsatisfactory performance, a score of 3 indicated moderate performance, and a score of 5 indicated fully satisfactory performance. A detailed description of the 5-point Likert scale is provided in Table 1.

The responses were independently scored by 4 senior spine surgeons with experience in VCF management (S2, with 31 years of experience; S3, with 25 years of experience; S4, with 22 years of experience; S5, with 26 years of experience) over a 2-week period. None of the 4 evaluators had prior experience in guideline development or formal experience in using LLMs for clinical guideline evaluation. Before scoring, researcher S1 preprocessed the evaluation materials. All nonanonymous information that might reveal the source of the answers, including model names, interface formatting, and any platform-generated disclaimers or warning messages, was removed. The order of questions and model responses was randomized. In addition, S1 recorded the section, question type, recommendation grade, and corresponding NASS recommendation for each question and compiled this information into a Microsoft Excel table (Multimedia Appendix 2). During scoring, the 4 evaluators consulted the underlying guideline text in the form of question-recommendation pairs and completed their independent ratings on this basis. Before the evaluation was conducted, all surgeons were required to thoroughly familiarize themselves with the evaluation checklist and guidelines. For the 2 potentially overlapping dimensions of self-awareness and fabrication and falsification, evaluators judged responses to demonstrate stronger self-awareness when the LLM explicitly acknowledged specific knowledge or evidentiary limitations. Generic uncertainty hedging alone was not scored as strong self-awareness. Responses were judged to involve fabrication and falsification when they contained unsupported, distorted, or falsely attributed content. Statements that remained broadly consistent with the overall direction of the guideline, were presented as inference rather than established recommendation, and did not fabricate evidence were not classified as fabrication and falsification. No formal pilot calibration or consensus-scoring session was conducted before independent rating. During the evaluation procedure, the surgeons were blinded to the source of the responses. The average score of the 4 surgeons represented the final score for each dimension, and interrater agreement was calculated to determine the reliability among surgeon evaluations. To determine whether the evaluators assigned the same absolute scores, rather than merely showing similar scoring trends, interrater agreement was evaluated using the intraclass correlation coefficient (ICC) with a 2-way mixed-effects model [30]. The ICC interpretations were as follows: excellent (ICC≥0.90); good (0.75≤ICC<0.90); moderate (0.50≤ ICC<0.75); and poor (ICC<0.50).

Subgroup analysis was performed according to question type (open-ended/closed-ended questions), recommendation grade (recommendation grades A-C/I), and VCF type (osteoporotic vertebral compression fracture [OVCF]/neoplastic vertebral compression fracture [NVCF]). Because open-ended questions were not always linked to a single recommendation grade, whereas recommendation grades were assigned to individual guideline recommendations, subgroup analysis by recommendation grade was performed only for closed-ended questions.

**Table 1.** Interpretation of the 5-point Likert scale system for evaluating LLM^a^ responses.

Evaluation items and Likert scale score	Interpretation
Accuracy^b^^,c^: Correctness of the LLM responses compared to the benchmarks	
5	Completely accurate
4	Accurate
3	Neutral
2	Inaccurate
1	Completely inaccurate
Consistency^b^^,c^: Stability and uniformity of the LLM responses to repeat questions	
5	Completely consistent
4	Consistent
3	Neutral
2	Inconsistent
1	Completely inconsistent
Self-awareness^b^^,c^: The ability of LLM to recognize its limitations and avoid overconfidence	
5	Perfectly self-aware
4	Self-aware
3	Neutral
2	Unaware
1	Extremely unaware
Fabrication and Falsification^b^^,c^: Existence of made-up or distorted information in the LLM responses	
5	Perfect
4	Acceptable
3	Neutral
2	Poor
1	Unacceptable
Comprehensiveness^c^: Completeness and coverage of the LLM responses to the prompts	
5	Extremely comprehensive
4	Comprehensive
3	Neutral
2	Incomplete
1	Extremely incomplete
Clarity^c^: Readability and intelligibility of the LLM responses for the readers	
5	Completely clear
4	Clear
3	Neutral
2	Unclear
1	Completely unclear
Trust and Confidence^c^: The degree of trust and confidence for users to LLM and its responses	
5	Complete trust and confidence
4	Trust and confidence
3	Neutral
2	Limited trust and confidence
1	No trust or confidence

a LLM: large language model.

b Evaluation items for closed-ended questions.

c Evaluation items for open-ended questions.

### Statistical Analysis

Statistical analysis was conducted using SPSS Statistics software (version 24.0; IBM Corp). The unit of analysis was the mean score assigned by the 4 evaluators to each model’s first response. Comparisons between LLMs across the evaluated dimensions based on the 5-point Likert scale were prespecified analyses, whereas subgroup analyses were considered exploratory. Due to the equidistance of the 5-point Likert scale, all data were expressed as mean (SD) to facilitate comparisons across dimensions and subgroups. The Mann-Whitney *U* test was used to analyze ranked data because it is a nonparametric method appropriate for ordinal data and does not rely on the assumption of normality. *P*<.05 was considered statistically significant.

## Results

**Figure 1.**
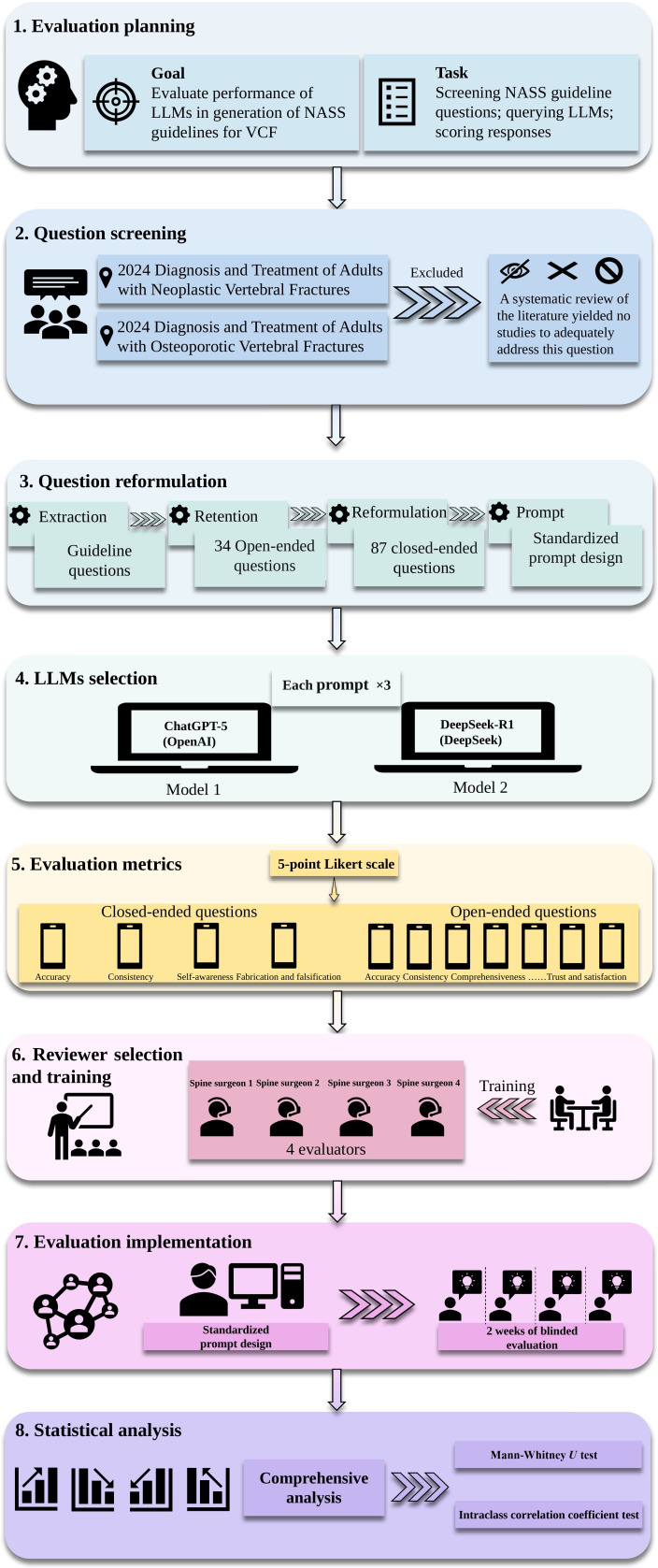
Flowchart of the cross-sectional study design for evaluating the performance of large language models on guidelines for adult vertebral compression fractures. LLM: large language model; NASS: North American Spine Society; VCF: vertebral compression fracture.

The LLMs generated a total of 726 responses (Multimedia Appendix 2), including 242 first-time responses. Interrater reliability was assessed for all evaluated dimensions, including 4 dimensions for closed-ended responses and 7 dimensions for open-ended responses. The ICCs ranged from 0.607 to 0.911, indicating moderate to excellent agreement (Table 2). The study flowchart is shown in Figure 1.

ChatGPT-5 and DeepSeek-R1 performed well overall. DeepSeek-R1 demonstrated significantly better consistency than ChatGPT-5 in both closed-ended (mean 4.08, SD 0.52 vs mean 3.76, SD 0.42; *P*<.001) and open-ended questions (mean 4.06, SD 0.51 vs mean 3.65, SD 0.34; *P*=.001). For open-ended questions, ChatGPT-5 showed significantly higher comprehensiveness than DeepSeek-R1 (mean 4.28, SD 0.64 vs mean 3.82, SD 0.89; *P*=.03), whereas DeepSeek-R1 achieved significantly higher trust and confidence scores (mean 4.35, SD 0.40 vs mean 4.03, SD 0.52; *P*=.02). In contrast, for closed-ended questions, the 2 models showed comparable performance in accuracy (mean 4.36, SD 0.70 vs mean 3.98, SD 1.04; *P*=.11), self-awareness (mean 3.87, SD 0.48 vs mean 3.93, SD 0.24; *P*=.10), and fabrication and falsification (mean 4.30, SD 0.35 vs mean 4.35, SD 0.51; *P*=.10). Likewise, for open-ended questions, no significant between-model differences were observed in accuracy (mean 3.74, SD 0.88 vs mean 3.89, SD 0.74; *P*=.42), self-awareness (mean 3.69, SD 0.65 vs mean 3.79, SD 0.23; *P*=.22), fabrication and falsification (mean 4.13, SD 0.45 vs mean 4.07, SD 0.57; *P*=.64), or clarity (mean 4.27, SD 0.60 vs mean 4.44, SD 0.35; *P*=.48; Table 3).

**Figure 2.**
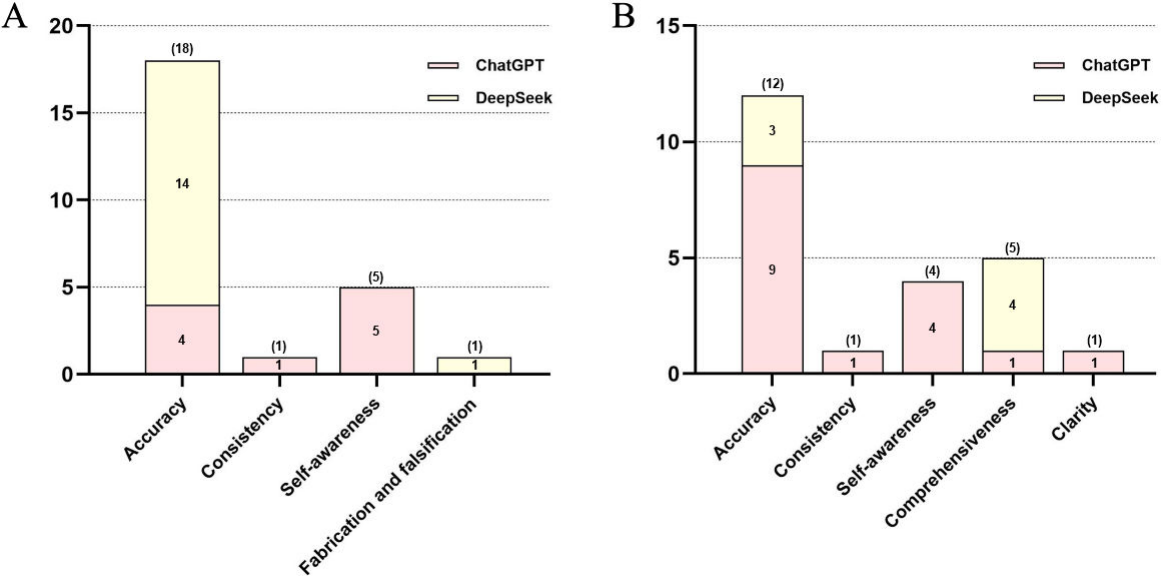
Distribution of low-performing response-subdimension occurrences by model and subdimension. (A) Closed-ended responses and (B) open-ended responses. The y-axis represents the number of occurrences of low-performing response subdimensions. Counts across subdimensions are not mutually exclusive.

Responses with low performance in at least 1 subdimension (score <3) were further identified and analyzed. Among the 242 first-time responses, 41 responses met this criterion. Of these, 23 were responses to closed-ended questions and 18 were responses to open-ended questions. When classified by LLM type, 22 were generated by ChatGPT-5 and 19 by DeepSeek-R1. These 41 low-performing first-time responses corresponded to 35 unique questions because both LLMs could generate low-performing responses to the same question, including 20 closed-ended and 15 open-ended questions. Among closed-ended responses, ChatGPT-5 generated 9 low-performing responses, with low performance in accuracy, consistency, self-awareness, and fabrication and falsification in 4, 1, 5, and 0 responses, respectively; DeepSeek-R1 generated 14 low-performing responses, with corresponding counts of 14, 0, 0, and 1. Among open-ended responses, ChatGPT-5 generated 13 low-performing responses, with low performance in accuracy, consistency, self-awareness, fabrication and falsification, comprehensiveness, clarity, and trust and confidence in 9, 1, 4, 0, 1, 1, and 0 responses, respectively; DeepSeek-R1 generated 5 low-performing responses, with corresponding counts of 3, 0, 0, 0, 4, 0, and 0. For question types, LLM responses to closed-ended questions showed low performance in accuracy (18/23, 78.26%) and self-awareness (5/23, 21.74%), while LLM responses to open-ended questions exhibited low performance in accuracy (12/18, 66.67%) and comprehensiveness (5/18, 27.78%). After distinguishing between LLM types, we observed that ChatGPT-5 demonstrated low subdimension performance in accuracy (13/22, 59.09%) and self-awareness (9/22, 40.91%), while DeepSeek-R1 showed low performance in accuracy (17/19, 89.47%) and comprehensiveness (4/19, 21.05%; Figure 2). Among the 35 unique original questions associated with low-performing responses, the most common sections were interventional treatment (16/35, 45.71%) and surgical treatment (8/35, 22.86%), while for recommendation grade, they were concentrated mainly in recommendation grade I (13/20, 65.00%). Moreover, ChatGPT-5 was more likely to generate unsatisfactory responses than DeepSeek-R1 when answering open-ended questions (13/18, 72.22%; 5/18, 27.78%), while the opposite was observed for closed-ended questions (9/23, 39.13%; 14/23, 60.87%).

**Table 2.** Interrater reliability across evaluation dimensions for open-ended and closed-ended questions.

Dimension	Open-ended questions, ICC^a^ (95% CI)	Closed-ended questions, ICC (95% CI)
Accuracy	0.911 (0.849‐0.951)	0.864 (0.803‐0.908)
Consistency	0.739 (0.563‐0.857)	0.745 (0.644‐0.823)
Self-awareness	0.830 (0.711‐0.907)	0.871 (0.817‐0.912)
Fabrication and falsification	0.776 (0.624‐0.877)	0.607 (0.453‐0.727)
Comprehensiveness	0.897 (0.793‐0.948)	—^b^
Clarity	0.754 (0.588‐0.865)	—
Trust and confidence	0.830 (0.628‐0.919)	—

a ICC: intraclass correlation coefficient.

b Not applicable.

**Table 3.** Performance comparison of ChatGPT-5 and DeepSeek-R1 on guideline-derived questions for adult vertebral compression fractures.^a^

Evaluation dimension	ChatGPT-5, mean (SD); median (IQR)	DeepSeek-R1, mean (SD); median (IQR)	*P* value
Closed-ended questions			
Accuracy	4.36 (0.70); 4.50 (4.25‐4.75)	3.98 (1.04); 4.25 (3.25‐5.00)	.11
Consistency	3.76 (0.42); 3.75 (3.50‐4.00)	4.08 (0.52); 4.00 (3.75‐4.50)	<.001^b^
Self-awareness	3.87 (0.48); 4.00 (4.00‐4.00)	3.93 (0.24); 4.00 (3.75‐4.00)	.10
Fabrication and falsification	4.30 (0.35); 4.25 (4.00‐4.50)	4.35 (0.51); 4.50 (4.00‐4.75)	.10
Open-ended questions			
Accuracy	3.74 (0.88); 4.00 (2.69‐4.25)	3.89 (0.74); 4.13 (3.19‐4.50)	.42
Consistency	3.65 (0.34); 3.75 (3.25‐4.00)	4.06 (0.51); 4.00 (3.75‐4.50)	.001^b^
Self-awareness	3.69 (0.65); 4.00 (3.25‐4.00)	3.79 (0.23); 3.75 (3.69‐4.00)	.22
Fabrication and falsification	4.13 (0.45); 4.25 (3.75‐4.50)	4.07 (0.57); 4.00 (3.50‐4.50)	.64
Comprehensiveness	4.28 (0.64); 4.38 (3.75‐5.00)	3.82 (0.89); 4.25 (3.25‐4.50)	.03^b^
Clarity	4.27 (0.60); 4.50 (4.19‐4.75)	4.44 (0.35); 4.50 (4.25‐4.75)	.48
Trust and confidence	4.03 (0.52); 4.00 (4.00‐4.50)	4.35 (0.40); 4.25 (4.00‐4.75)	.02^b^

a Comparisons between ChatGPT-5 and DeepSeek-R1 across evaluation dimensions were prespecified analyses.

b Statistically significant differences at *P*<.05.

Subgroup analysis was performed according to question type (open-ended or closed-ended questions), recommendation grade (recommendation grades A-C/I), and VCF type (OVCF/NVCF). An overall better performance was noted for responses to closed-ended questions than for open-ended ones. The responses to closed-ended questions were superior to those of open-ended questions in accuracy (mean 4.17, SD 0.90 vs mean 3.81, SD 0.81; *P*<.001), self-awareness (mean 3.90, SD 0.38 vs mean 3.74, SD 0.48; *P*<.001), and fabrication and falsification (mean 4.32, SD 0.44 vs mean 4.10, SD 0.51; *P*=.001) but not in consistency (mean 3.92, SD 0.50 vs mean 3.85, SD 0.48; *P*=.14). The responses to the questions with recommendation grades A-C outperformed those with grade I in accuracy (mean 4.51, SD 0.75 vs mean 3.80, SD 0.92; *P*<.001), consistency (mean 4.02, SD 0.46 vs mean 3.80, SD 0.52; *P*=.001), and fabrication and falsification (mean 4.54, SD 0.32 vs mean 4.09, SD 0.43; *P*<.001) but showed lower self-awareness (mean 3.82, SD 0.39 vs mean 3.99, SD 0.35; *P*<.001). No statistically significant differences were observed between VCF subtypes (Table 4).

**Figure 3.**
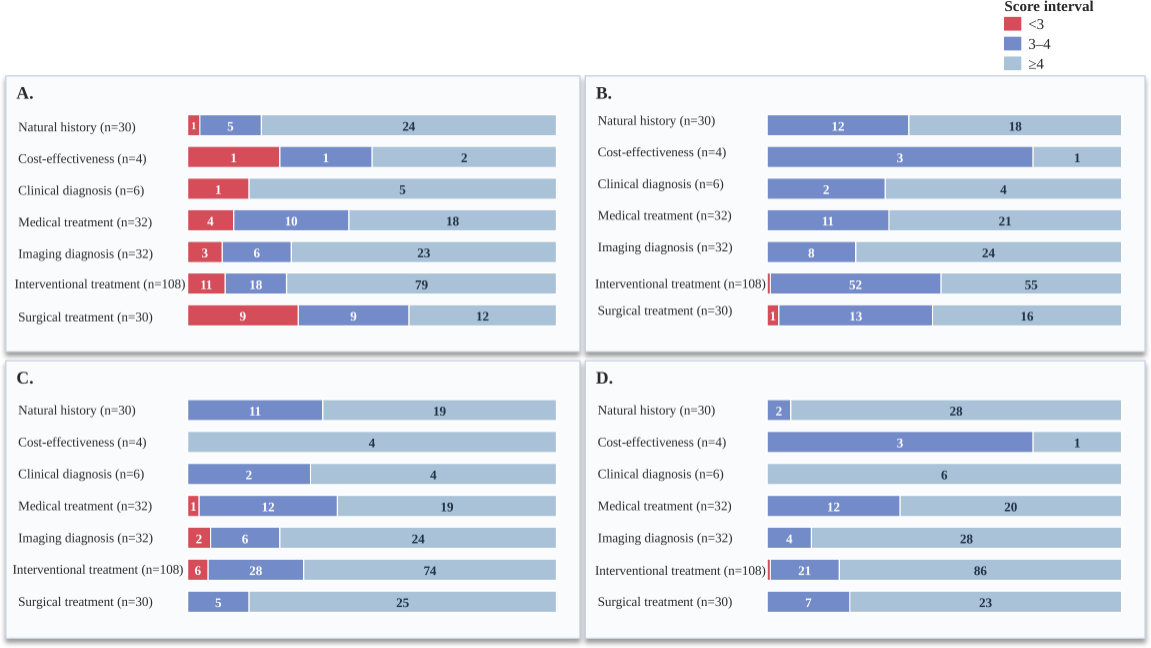
Stacked bar charts showing the distribution of score intervals across clinical sections for 4 key evaluation dimensions. (A) Accuracy, (B) consistency, (C) self-awareness, and (D) fabrication and falsification. Each bar represents the proportion of responses within a clinical section that fell into the score intervals of ≥4, 3‐4, and <3. In each section label, n indicates the number of evaluated first-time responses rather than the number of unique clinical questions.

Stacked bar charts visualize the influence of sections on scores (Figure 3). The vast majority of sections scored ≥4 in most dimensions, particularly in self-awareness (169/242, 69.8%) and fabrication and falsification (192/242, 79.3%). The sections of natural history and clinical diagnosis demonstrated outstanding performance in accuracy and fabrication and falsification, with over 80.0% achieving scores of ≥4. Likewise, cost-effectiveness and surgical treatment stood out in self-awareness, while imaging diagnosis was prominent in fabrication and falsification. However, all sections were more likely to generate unsatisfactory responses (score <3) in accuracy, particularly in surgical treatment, which reached a high proportion of 30% and warranted further attention.

**Table 4.** Subgroup analysis of large language model performance according to question type, recommendation grade, and vertebral compression fracture subtype.^a^

Evaluation dimension	Open-ended questions (n=68), mean (SD); median (IQR)	Closed-ended questions (n=174), mean (SD); median (IQR)	*P* value	Recommendation grade A-C (n=92), mean (SD); median (IQR)^b^	Recommendation grade I (n=82), mean (SD); median (IQR)^b^	*P* value	OVCF^c^ (n=182), mean (SD); median (IQR)	NVCF^d^ (n=60), mean (SD); median (IQR)	*P* value
Accuracy	3.81 (0.81); 4.00 (3.06‐4.50)	4.17 (0.90); 4.50 (3.50‐4.75)	<.001^e^	4.51 (0.75); 4.75 (4.50‐5.00)	3.80 (0.92); 4.00 (3.25‐4.50)	<.001^e^	4.05 (0.89); 4.25 (3.50‐4.75)	4.14 (0.89); 4.50 (3.50‐4.75)	.33
Consistency	3.85 (0.48); 3.75 (3.50‐4.00)	3.92 (0.50); 4.00 (3.50‐4.25)	.14	4.02 (0.46); 4.00 (3.75‐4.25)	3.80 (0.52); 3.88 (3.25‐4.00)	.001^e^	3.88 (0.50); 4.00 (3.50‐4.25)	3.98 (0.48); 4.00 (3.75‐4.25)	.21
Self-awareness	3.74 (0.48); 3.88 (3.50‐4.00)	3.90 (0.38); 4.00 (4.00‐4.00)	<.001^e^	3.82 (0.39); 4.00 (3.75‐4.00)	3.99 (0.35); 4.00 (4.00‐4.06)	<.001^e^	3.84 (0.44); 4.00 (3.75‐4.00)	3.92 (0.33); 4.00 (3.75‐4.00)	.45
Fabrication and falsification	4.10 (0.51); 4.25 (3.75‐4.50)	4.32 (0.44); 4.50 (4.00‐4.75)	.001^e^	4.54 (0.32); 4.50 (4.50‐4.75)	4.09 (0.43); 4.13 (3.75‐4.50)	<.001^e^	4.26 (0.47); 4.25 (4.00‐4.50)	4.28 (0.47); 4.38 (3.81‐4.75)	.84
Comprehensiveness^f^	—^g^	—	—	—	—	—	4.06 (0.79); 4.25 (3.50‐4.75)	4.03 (0.85); 4.25 (3.75‐4.50)	.87
Clarity^f^	—	—	—	—	—	—	4.35 (0.52); 4.50 (4.25‐4.75)	4.36 (0.43); 4.50 (4.06‐4.75)	.93
Trust and confidence^f^	—	—	—	—	—	—	4.25 (0.51); 4.38 (4.00‐4.50)	4.05 (0.43); 4.00 (4.00‐4.44)	.07

a The subgroup comparisons in this table were exploratory analyses. n indicates the number of evaluated first-time responses included in each subgroup rather than the number of unique clinical questions.

b Comparison between responses based on recommendation grade was conducted only in closed-ended questions.

c OVCF: osteoporotic vertebral compression fracture.

d NVCF: neoplastic vertebral compression fracture.

e Statistically significant differences are indicated by *P*<.05.

f Comparison in these dimensions only conducted in open-ended questions (OVCF: n=48; NVCF: n=20).

g Not applicable.

## Discussion

### Principal Findings

VCFs have become an increasingly serious global public health problem, which may result in serious clinical consequences and significantly affect patients’ quality of life [4]. Their prevention and treatment depend on multidisciplinary integrated management, and a comprehensive grasp of evaluation, diagnosis, treatment, and long-term management strategies is essential to reduce complications [10,31,32]. Evidence-based clinical practice guidelines provide robust support for standardized diagnosis and treatment. However, they are characterized by a lengthy format, complex information, regional variations, and periodic updates, limiting their efficient application in clinical practice. In this context, artificial intelligence (AI) technology with rapid information integration and real-time updating capabilities offers clinicians and patients a new avenue for dynamically accessing authoritative recommendations. This study was the first to systematically evaluate the performance of DeepSeek-R1 and ChatGPT-5 for VCF guideline questions. The 2 models showed generally high but variable scores across evaluation dimensions, with residual deficiencies particularly in interventional and surgical treatment recommendations and in questions linked to recommendation grade I. This provides a preliminary reference for clinicians to understand the practical value of LLMs in responding to questions related to the latest VCF guidelines.

### Comparison With Prior Work

Previous studies have used multilabel qualitative evaluation methods to investigate the performance of LLMs in answering NASS guideline questions across dimensions such as accuracy, overconclusiveness, supplementary information, and incompleteness [16,33]. The results indicated that LLMs could provide relatively accurate and reasonable medical advice, demonstrating promising potential for application in clinical decision support. Nevertheless, given their tendency to generate ambiguous or imprecise responses, the advantages of quantitative scoring systems have been emphasized and leveraged [34-37]. Therefore, this study conducted a multidimensional quantitative assessment of LLMs using the 5-point Likert scale. Although the overall accuracy of the 2 models was acceptable, the proportion of low-quality responses remained relatively high. Furthermore, Figure 3 suggested that nearly all sections were more prone to generating poor responses (score <3) in accuracy, particularly in surgical treatment. These errors tended to follow several recurring patterns. In some cases, guideline statements indicating insufficient evidence were transformed into overly definitive claims. In addition, some responses introduced overly prescriptive treatment implications beyond the evidentiary scope of the guideline, whereas others incompletely synthesized broad open-ended surgical questions (Multimedia Appendix 3). Consistent with previous studies, this finding indicated that LLMs still encountered significant limitations when processing treatment recommendations involving the latest medical knowledge and complex clinical experience [38]. Guidelines generally avoid making explicit recommendations on controversial questions, but LLMs tend to generate more assertive answers, which is a double-edged sword. Furthermore, the stability of model outputs is critical to clinical practice, as occasional errors may cause serious consequences [35,39,40]. Notably, the models in this study exhibited a high degree of consistency, with DeepSeek-R1 outperforming ChatGPT-5 in this dimension. In summary, LLMs demonstrate acceptable capability in generating VCF guideline information, highlighting their potential to assist clinicians by reducing the burden of information retrieval and decision-making. However, the technical performance of LLMs does not necessarily translate into clinical suitability. Although their mean accuracy approached 4/5, even a small number of hallucinations or other errors at critical decision points in high-risk domains, such as interventional and surgical treatment, could still lead to unsafe clinical decisions. Therefore, even when the overall mean accuracy of LLMs appears relatively high, they may still be unsuitable for unsupervised clinical use in such settings. Accordingly, further optimization and validation are still needed to improve their reliability in specific high-difficulty and high-risk tasks.

Another crucial dimension for evaluating the application potential of LLMs is safety, including self-awareness and fabrication and falsification [20]. Self-awareness reflects a model’s capacity to recognize the limitations of its data sources, processing mechanisms, and knowledge. We noted that DeepSeek prominently displayed the following message at the bottom of its interface: “This response is AI-generated, for reference only,” advising users to exercise caution when using the generated content. This study found that both LLMs demonstrated moderate levels of self-awareness. Previous studies suggested that most LLM-generated responses exhibited some level of self-awareness, but this simultaneously undermined user confidence in these models. Future iterations must achieve a balance between response accuracy and self-awareness [37,39]. In contrast, both models performed excellently in fabrication and falsification (score >4), demonstrating high reliability in avoiding fictional or distorted information.

To further investigate the influence of question types on LLM performance, differences between open-ended and closed-ended questions were compared across 4 key dimensions. Previous studies primarily focused on closed-ended questions, which struggled to capture the complexity of medical decision-making. Moreover, the performance of LLMs on different types of questions is debatable. Goodman et al [41] reported no significant difference in LLMs’ performance when answering descriptive vs binary medical questions. However, Zaidat et al [15] and Zhang et al [29] found that LLMs performed better on closed-ended questions, consistent with our findings. This difference may be attributed to the inherent characteristics of the questions themselves. Open-ended questions are characterized by their broad scope, ambiguous wording, and the involvement of complex factors, easily leading to the omission of critical information. Conversely, closed-ended questions are clearer and more specific, enabling LLMs to understand them more accurately, aligning with current prompt design principles [24-26].

Another notable concern is the influence of recommendation grade on response quality. Previous studies revealed that ChatGPT provided more accurate responses to guideline questions supported by clinical evidence than to those with insufficient or conflicting evidence [11,18], which was consistent with our results. However, in the dimension of self-awareness, the models’ responses to questions with recommendation grades A-C were inferior to those with recommendation grade I, reflecting progress in the models’ ability to exercise self-restraint in the absence of evidence-based support. These findings suggest that the accuracy of LLM responses is not always aligned with the strength of the underlying evidence and that safer deployment may require system-level safeguards, such as uncertainty labels that explicitly reflect recommendation grade and direct links to the relevant guideline text [42]. Furthermore, although we initially anticipated that LLMs might generate lower-quality responses to NVCF compared to OVCF due to its relative rarity and complex treatment, the results encouragingly showed no significant differences. We speculate that this may be attributable to the recently published NVCF guidelines and related appropriateness criteria, which provide more refined evidence-based recommendations, as well as to the broad availability of high-quality training data, suggesting that current LLMs have the potential to comprehensively answer VCF guideline questions.

Moreover, further evaluation of open-ended questions revealed that ChatGPT-5 demonstrated superior performance in comprehensiveness, whereas DeepSeek-R1 showed advantages in consistency and trust and confidence. This suggests that, in practice, DeepSeek-R1 may be better suited for standardized clinical pathways where reproducibility is critical, whereas ChatGPT-5 may offer an advantage when broader explanatory coverage is needed for open-ended, clinician-oriented queries. Given that no single LLM is currently suitable for all scenarios, research should involve testing and comparing multiple LLMs to understand their strengths and weaknesses in specific tasks. This study highlighted the importance of users considering query requirements, question types, and model performance when using LLMs. Another point that merits attention is that the findings of this study were obtained under a standardized clinician-oriented role-prompting condition, rather than under naïve prompts, patient-style queries, or real-world queries embedded in routine clinician workflow. Compared with the structured role prompt used in this study, naïve prompts may yield lower or more unstable performance because they provide less professional framing and fewer cues to support evidence-based responses. Patient-style queries may further alter performance because they often introduce greater ambiguity and less precise terminology [43]. Clinician workflow-based queries may also differ from our study setting because real-world clinical use typically involves richer case context, iterative clarification, and repeated questioning rather than a single isolated prompt [44]. In addition, no adjustment for multiple comparisons was applied to the pairwise comparisons across evaluation dimensions and subgroups. Therefore, caution is warranted when interpreting the subgroup findings and when extrapolating the conclusions of this study to other prompting scenarios, other guideline questions, or different LLMs.

Beyond technical performance, the ethical and governance implications of medical LLM deployment also warrant explicit consideration. First, the limited transparency of current commercial LLMs constrains independent verification of how outputs are generated and whether they remain aligned with evolving evidence. Second, because our evaluation was based on guideline-derived questions, it necessarily emphasized guideline concordance and may not have fully captured other clinically important perspectives, such as patient preferences, multimorbidity, and potential disagreement across evidence sources. Third, when LLM outputs diverge from source guidelines, questions of accountability and appropriate human oversight become especially important [45]. Different use cases imply different safety expectations and regulatory requirements. Based on the present findings, we consider current LLMs to be more appropriately positioned as adjunctive reference tools for rapid guideline navigation by clinicians, while final clinical decisions must remain the responsibility of qualified clinicians. In the future, the broader integration of LLMs into health care systems should be grounded in improved model transparency, reduced data bias, protection of data privacy, and strict ethical oversight and accountability mechanisms to ensure their safe and sustainable incorporation into clinical practice.

### Limitations and Future Directions

There are some limitations present in this study. First, despite the rigorous study design, potential biases may still exist due to the limited number of models, evaluators, and questions. Second, given the rapid iteration of AI technology, the findings of this cross-sectional study reflected only the models’ performance at a particular point in time. As model training data continue to expand and model versions are continually updated, the findings of this study may not remain applicable to newer models; therefore, timely repeated cross-sectional evaluations will be necessary. Third, although prompt design drew upon relevant tutorials and guidelines, it might not fully unlock the optimal performance of LLMs. Furthermore, the structure and phrasing of the guidelines themselves may introduce inherent biases, thereby affecting the quality of responses. Accordingly, the performance observed under the prompting strategy used in this study may underestimate the best achievable outputs of the models. Future studies should compare multiple prompting approaches, including more tailored and model-specific strategies as well as iterative prompting methods. Fourth, this study focused on evaluating the potential of DeepSeek-R1 and ChatGPT-5 in assisting medical decision-making and providing professional information, without exploring their performance in other clinical scenarios. Fifth, although all evaluators were required to familiarize themselves with the evaluation checklist and guideline content before scoring, no formal pilot calibration or consensus-building phase was conducted prior to independent assessment. This may have allowed interrater differences in more subjective dimensions and, in turn, affected scoring reliability. In addition, all evaluators were spine surgeons from the same institution, which may have introduced specialty bias and institution-specific interpretive bias. Future studies should include multidisciplinary evaluators from different institutions, together with a calibration phase incorporating pilot scoring and consensus discussion, to improve interrater consistency and mitigate specialty bias. Sixth, although the memory setting and internet search were disabled, repeated prompts for each question-model pair were entered in separate new sessions, and no custom instructions were used; the study was still conducted through a consumer-facing web interface rather than an application programming interface–pinned workflow. Therefore, while account-level carryover was likely minimized, exact execution-level reproducibility could not be fully guaranteed because backend-level or product-level updates may have affected model behavior over time. Future studies should consider application programming interface–based evaluation pipelines with pinned model snapshots to further strengthen reproducibility and version transparency [46]. Seventh, the main analyses in this study were based on question-level mean scores, and hierarchical dependence at the question or rater level was not explicitly modeled. Future studies could use mixed-effects approaches to account more formally for the multilevel data structure. Finally, we evaluated only 2 LLMs that were easily accessible and widely used among the public. Many experimental and domain-specific LLMs were excluded owing to the unavailability of their architectures or user interfaces. This limits the generalizability of the conclusions of this study to other LLMs. Future evaluation studies should include a broader range of LLMs and clinical questions. Despite these limitations, this study not only improved our understanding of the capabilities and applicability of LLMs in the field of VCF but also provided valuable insights for the subsequent exploration of more advanced LLMs in health care applications.

### Conclusion

Under a standardized clinician-oriented role-prompting condition, ChatGPT-5 and DeepSeek-R1 achieved generally high but variable scores on NASS VCF guideline-derived questions, with residual deficiencies in interventional and surgical treatment recommendations and questions linked to recommendation grade I. These findings should be interpreted within the controlled prompting setting used in this study, and caution is warranted when generalizing them to other prompting scenarios, patient-style queries, clinician workflow–based real-world queries, or different LLMs.

## Supplementary material

10.2196/87816 Multimedia Appendix 1 Main characteristics of the evaluated large language models.

10.2196/87816 Multimedia Appendix 2 Guideline-derived question set and anonymized large language model responses.

10.2196/87816 Multimedia Appendix 3 Examples of low-performing responses and dominant error patterns.

10.2196/87816 Checklist 1 STROBE checklist.

## Abbreviations

AI: artificial intelligence
ICC: intraclass correlation coefficient
LLM: large language model
NASS: North American Spine Society
NVCF: neoplastic vertebral compression fracture
OVCF: osteoporotic vertebral compression fracture
QUEST: Quality of Information, Understanding and Reasoning, Expression Style and Persona, Safety and Harm, and Trust and Confidence
STROBE: Strengthening the Reporting of Observational Studies in Epidemiology
VCF: vertebral compression fracture

## References

[1] Burge R, Dawson-Hughes B, Solomon DH, Wong JB, King A, Tosteson A (2007). Incidence and economic burden of osteoporosis-related fractures in the United States, 2005-2025. J Bone Miner Res.

[2] Hoyt D, Urits I, Orhurhu V (2020). Current concepts in the management of vertebral compression fractures. Curr Pain Headache Rep.

[3] Kim HJ, Park S, Park SH (2018). Prevalence of frailty in patients with osteoporotic vertebral compression fracture and its association with numbers of fractures. Yonsei Med J.

[4] Parreira PCS, Maher CG, Megale RZ, March L, Ferreira ML (2017). An overview of clinical guidelines for the management of vertebral compression fracture: a systematic review. Spine J.

[5] Wang H, Sribastav SS, Ye F (2015). Comparison of percutaneous vertebroplasty and balloon kyphoplasty for the treatment of single level vertebral compression fractures: a meta-analysis of the literature. Pain Physician.

[6] Varacallo MA, Fox EJ (2014). Osteoporosis and its complications. Med Clin North Am.

[7] Oleksik A, Lips P, Dawson A (2000). Health-related quality of life in postmenopausal women with low BMD with or without prevalent vertebral fractures. J Bone Miner Res.

[8] Oleksik AM, Ewing S, Shen W, van Schoor NM, Lips P (2005). Impact of incident vertebral fractures on health related quality of life (HRQOL) in postmenopausal women with prevalent vertebral fractures. Osteoporos Int.

[9] Wong CC, McGirt MJ (2013). Vertebral compression fractures: a review of current management and multimodal therapy. J Multidiscip Healthc.

[10] Alsoof D, Anderson G, McDonald CL, Basques B, Kuris E, Daniels AH (2022). Diagnosis and management of vertebral compression fracture. Am J Med.

[11] Wang T, Chen R, Wang B (2025). Evaluating the performance of state-of-the-art artificial intelligence chatbots based on the WHO global guidelines for the prevention of surgical site infection: cross-sectional study. J Med Internet Res.

[12] Zhao B, Liu H, Liu Q (2025). Breaking boundaries in spinal surgery: GPT-4’s quest to revolutionize surgical site infection management. J Infect Dis.

[13] Bernstein IA, Zhang YV, Govil D (2023). Comparison of ophthalmologist and large language model chatbot responses to online patient eye care questions. JAMA Netw Open.

[14] Connors C, Gupta K, Khusid JA (2024). Evaluation of the current status of artificial intelligence for endourology patient education: a blind comparison of ChatGPT and Google Bard against traditional information resources. J Endourol.

[15] Zaidat B, Shrestha N, Rosenberg AM (2024). Performance of a large language model in the generation of clinical guidelines for antibiotic prophylaxis in spine surgery. Neurospine.

[16] Duey AH, Nietsch KS, Zaidat B (2023). Thromboembolic prophylaxis in spine surgery: an analysis of ChatGPT recommendations. Spine J.

[17] Shrestha N, Shen Z, Zaidat B (2024). Performance of ChatGPT on NASS clinical guidelines for the diagnosis and treatment of low back pain: a comparison study. Spine (Phila Pa 1976).

[18] Ahmed W, Saturno M, Rajjoub R (2024). ChatGPT versus NASS clinical guidelines for degenerative spondylolisthesis: a comparative analysis. Eur Spine J.

[19] Hoang T, Liou L, Rosenberg AM (2024). An analysis of ChatGPT recommendations for the diagnosis and treatment of cervical radiculopathy. J Neurosurg Spine.

[20] Tam TYC, Sivarajkumar S, Kapoor S (2024). A framework for human evaluation of large language models in healthcare derived from literature review. NPJ Digit Med.

[21] von Elm E, Altman DG, Egger M (2007). Strengthening the Reporting of Observational Studies in Epidemiology (STROBE) statement: guidelines for reporting observational studies. BMJ.

[22] (2024). Evidence-based clinical guidelines multidisciplinary spine care: diagnosis and treatment of adults with osteoporotic vertebral compression fractures. https://www.spine.org/Portals/0/assets/downloads/ResearchClinicalCare/Guidelines/Osteoporotic-Vertebral-Compression-Fractures.pdf.

[23] (2024). Evidence-based clinical guidelines for multidisciplinary spine care: diagnosis and treatment of adults with neoplastic vertebral fractures. https://www.spine.org/Portals/0/assets/downloads/ResearchClinicalCare/Guidelines/Neoplastic-Vertebral-Fractures.pdf.

[24] Meskó B (2023). Prompt engineering as an important emerging skill for medical professionals: tutorial. J Med Internet Res.

[25] Pu Z, Shi CL, Jeon CO (2024). ChatGPT and generative AI are revolutionizing the scientific community: a Janus-faced conundrum. Imeta.

[26] Maaz S, Palaganas JC, Palaganas G, Bajwa M (2024). A guide to prompt design: foundations and applications for healthcare simulationists. Front Med (Lausanne).

[27] Wu J, Wang Z, Qin Y (2025). Performance of DeepSeek-R1 and ChatGPT-4o on the Chinese national medical licensing examination: a comparative study. J Med Syst.

[28] Chan L, Xu X, Lv K (2025). DeepSeek-R1 and GPT-4 are comparable in a complex diagnostic challenge: a historical control study. Int J Surg.

[29] Zhang L, Wang T, Zheng Y, Kong X, Hong G, Zang L (2025). Assessment of ChatGPT’s adherence to evidence-based clinical practice guidelines for plantar fasciitis management. J Orthop Surg Res.

[30] Koo TK, Li MY (2016). A guideline of selecting and reporting intraclass correlation coefficients for reliability research. J Chiropr Med.

[31] McCarthy J, Davis A (2016). Diagnosis and management of vertebral compression fractures. Am Fam Physician.

[32] Goldstein CL, Chutkan NB, Choma TJ, Orr RD (2015). Management of the elderly with vertebral compression fractures. Neurosurgery.

[33] Mejia MR, Arroyave JS, Saturno M (2024). Use of ChatGPT for determining clinical and surgical treatment of lumbar disc herniation with radiculopathy: a North American Spine Society guideline comparison. Neurospine.

[34] Gianola S, Bargeri S, Castellini G (2024). Performance of ChatGPT compared to clinical practice guidelines in making informed decisions for lumbosacral radicular pain: a cross-sectional study. J Orthop Sports Phys Ther.

[35] Walker HL, Ghani S, Kuemmerli C (2023). Reliability of medical information provided by ChatGPT: assessment against clinical guidelines and patient information quality instrument. J Med Internet Res.

[36] Nwachukwu BU, Varady NH, Allen AA (2025). Currently available large language models do not provide musculoskeletal treatment recommendations that are concordant with evidence-based clinical practice guidelines. Arthroscopy.

[37] Sciberras M, Farrugia Y, Gordon H (2024). Accuracy of information given by ChatGPT for patients with inflammatory bowel disease in relation to ECCO guidelines. J Crohns Colitis.

[38] Pan Y, Tian S, Guo J, Cai H, Wan J, Fang C (2025). Clinical feasibility of AI Doctors: evaluating the replacement potential of large language models in outpatient settings for central nervous system tumors. Int J Med Inform.

[39] Scaff SPS, Reis FJJ, Ferreira GE, Jacob MF, Saragiotto BT (2025). Assessing the performance of AI chatbots in answering patients’ common questions about low back pain. Ann Rheum Dis.

[40] Thorp HH (2023). ChatGPT is fun, but not an author. Science.

[41] Goodman RS, Patrinely JR, Stone CA (2023). Accuracy and reliability of chatbot responses to physician questions. JAMA Netw Open.

[42] Zhou S, Wang J, Xu Z (2025). Uncertainty-aware large language models for explainable disease diagnosis. NPJ Digit Med.

[43] Ye C, Zweck E, Ma Z, Smith J, Katz S (2024). Doctor versus artificial intelligence: patient and physician evaluation of large language model responses to rheumatology patient questions in a cross-sectional study. Arthritis Rheumatol.

[44] Artsi Y, Sorin V, Glicksberg BS, Korfiatis P, Nadkarni GN, Klang E (2025). Large language models in real-world clinical workflows: a systematic review of applications and implementation. Front Digit Health.

[45] (2024). Ethics and governance of artificial intelligence for health: guidance on large multi-modal models. https://iris.who.int/server/api/core/bitstreams/e9e62c65-6045-481e-bd04-20e206bc5039/content.

[46] Park SH, Suh CH, Lee JH (2025). Minimum reporting items for CLEAR evaluation of accuracy reports of large language models in healthcare (MI-CLEAR-LLM): 2025 updates. Korean J Radiol.

