# Supplementary material for: Performance of DeepSeek-R1 and ChatGPT-5 in the Generation of North American Spine Society Clinical Guidelines for Adult Vertebral Compression Fractures: Comparative Study
Source: J Med Internet Res. 2026 Jul 10;28:e87816. doi: 10.2196/87816 (PMC13353910; doi:10.2196/87816)
Supplement: Multimedia Appendix 1 [file jmir-v28-e87816-s001.docx]

**Multimedia Appendix 1.** The main features of the two LLMs used in this study.

**ChatGPT-5 (*OpenAI*)**

ChatGPT-5 was released on August 7, 2025. Manufacturers claim that ChatGPT-5 is their smartest, fastest, most useful model yet, with built-in thinking that puts expert-level intelligence in everyone’s hands. ChatGPT-5 is a significant leap in intelligence over all previous models, featuring state-of-the-art performance across coding, math, writing, health, visual perception, and more. It is a unified system that knows when to respond quickly and when to think longer to provide expert-level responses. ChatGPT‑5 is the best model yet for health-related questions, empowering users to be informed about and advocate for their health. The model also now provides more precise and reliable responses, adapting to the user’s context, knowledge level, and geography, enabling it to provide safer and more helpful responses in a wide range of scenarios. ChatGPT‑5 not only outperforms previous models on benchmarks and answers questions more quickly, but—most importantly—is more useful for real-world queries. Manufacturers claim that they have made significant advances in reducing hallucinations, improving instruction following, and minimizing sycophancy. Notably, manufacturers have particularly invested in making their models more reliable when reasoning on complex, open-ended questions. [1]

**DeepSeek-R1 (*DeepSeek*)**

DeepSeek-R1 has completed a minor version upgrade and the current version is DeepSeek-R1-0528, released on May 28, 2025. DeepSeek-R1-0528 still uses the DeepSeek-V3 Base model released in December 2024 as a base, but invests more computational power in the post-training process, which significantly improves the model's depth of thought and inference. The updated model has achieved excellent performance in a number of benchmarks, including math, programming and general logic, and is approaching the overall performance of other top international models, such as OpenAI o3 and Gemini-2.5-Pro. Compared to the previous version of DeepSeek-R1, the performance of the new version in complex reasoning tasks has been significantly improved. Furthermore, the new version of DeepSeek-R1 has been optimized for the “artificial hallucination” problem, and its hallucination rate has been reduced by about 45-50% compared with the old version, which can effectively provide more accurate and reliable results. [2]

**References**

1. Introducing GPT-4.5. Published February 27, 2025. Accessed July 10, 2025. [https://openai.com/index/introducing-gpt-5/]
2. DeepSeek-R1 updated for deeper thinking and stronger reasoning. Published May 28, 2025. Accessed July 10, 2025. [https://api-docs.deepseek.com/zh-cn/news/news250528]
